# The Effect of an Alternate Start Codon on Heterologous Expression of a PhoA Fusion Protein in *Mycoplasma gallisepticum*

**DOI:** 10.1371/journal.pone.0127911

**Published:** 2015-05-26

**Authors:** Indu S. Panicker, Glenn F. Browning, Philip F. Markham

**Affiliations:** Asia-Pacific Centre for Animal Health, Faculty of Veterinary and Agricultural Sciences, The University of Melbourne, Melbourne, Victoria, Australia; Miami University, UNITED STATES

## Abstract

While the genomes of many *Mycoplasma* species have been sequenced, there are no collated data on translational start codon usage, and the effects of alternate start codons on gene expression have not been studied. Analysis of the annotated genomes found that ATG was the most prevalent translational start codon among *Mycoplasma* spp. However in *Mycoplasma gallisepticum* a GTG start codon is commonly used in the *vlh*A multigene family, which encodes a highly abundant, phase variable lipoprotein adhesin. Therefore, the effect of this alternate start codon on expression of a reporter PhoA lipoprotein was examined in *M*. *gallisepticum*. Mutation of the start codon from ATG to GTG resulted in a 2.5 fold reduction in the level of transcription of the *pho*A reporter, but the level of PhoA activity in the transformants containing *pho*A with a GTG start codon was only 63% of that of the transformants with a *pho*A with an ATG start codon, suggesting that GTG was a more efficient translational initiation codon. The effect of swapping the translational start codon in *pho*A reporter gene expression was less in *M*. *gallisepticum* than has been seen previously in *Escherichia coli* or *Bacillus subtilis*, suggesting the process of translational initiation in mycoplasmas may have some significant differences from those used in other bacteria. This is the first study of translational start codon usage in mycoplasmas and the impact of the use of an alternate start codon on expression in these bacteria.

## Introduction

Although the number of genes involved in cellular metabolism have been reduced in mycoplasmas, the number of genes involved in transcription and translation are comparable to those of other bacteria [1, 2], and the basic mechanisms of transcription and translation in mycoplasmas are thought to be similar to those of low G+C Gram-positive bacteria. Translation is a complex process that involves four phases—initiation, elongation, termination and ribosome recycling [3]. Translational initiation in bacteria is influenced by a range of factors, including the ribosomal binding site (RBS) [4], mRNA secondary structure near the ribosomal binding site [5], the translational start codon [6] and the sequence downstream of the start codon [7]. In the *E*. *coli* K-12 genome, of the 4288 open reading frames annotated, 82% have an ATG start codon, 14.3% a GTG start codon and 3% a TTG start codon [8]. A recent annotation of ten different *E*. *coli* strains found that 82.5% of the start codons were ATG, 12.3% were GTG and 5% were TTG, with CTG, ATT and ATC used at lower frequencies [9]. Translational efficiency has been shown to decrease in *E*. *coli* when a start codon other than ATG is used, with an eight-fold reduction in translation seen with GTG or TTG start codons [10]. In *B*. *subtilis* ATG, TTG and GTG start codons are used in 78%, 13% and 9% of CDSs, respectively [11], with GTG shown to be three- to five-fold less efficient than ATG in translational initiation [12].

Mollicutes generally have genomes with a low G+C content and, as a result, an A or U(T) bias in codon usage, especially in the first and third codon positions. While the coding region of most mycoplasma genes begins with an ATG start codon, alternative start codons, such as GTG and TTG, are used [13]. In the genome of *M*. *gallisepticum* R_low_, based on *in silico* prediction of coding sequences, ATG is the preferred start codon, followed by GTG and TTG (http://services.cbib.u-bordeaux2.fr/molligen/). However, all the genes in the *vlh*A multigene family, which encodes the abundant phase variable lipoprotein adhesion VlhA, have a GTG translational initiation codon. The high prevalence of an atypical translational initiation codon in gene family encoding a highly expressed lipoprotein in a species with an overall bias against such GC rich codons suggests that this codon may not have the adverse effects on translation of the *vlhA* genes in *M*. *gallisepticum* that are seen in other genes in other bacterial species. In order to explore whether use of a GTG translational initiation codon was more common in mycoplasmas than in other bacterial species, we initially examined the annotated genomes of a number of *Mycoplasma* species to determine the prevalence of use of alternative translational start codons in predicted coding sequences in this family of bacteria. At present the genomes of 52 *Mycoplasma* species have been annotated, but there are no collated data on start codon usage in mycoplasmas. We then assessed the effect of the GTG translational start codon on transcription and translation of a reporter lipoprotein gene in *M*. *gallisepticum*.

## Methods

### Bacterial strains and culture conditions

*M*. *gallisepticum* strain S6 was grown in mycoplasma broth or on mycoplasma agar at 37°C, with 16 μg gentamicin (Invitrogen)/ml included in the media to select for transformants. *E*. *coli* DH5α cells were used as the host for genetic manipulation and cloning of plasmids.

### Construction of plasmids

#### Plasmid *ltufacypho*A (pTAP)

The promoter region of the gene of elongation factor Tu (*ltuf*) of *M*. *gallisepticum*, the leader sequence and acylation sequence of the *vlh*A1.1 gene from the genomic DNA of *M*. *gallisepticum* strain S6 and the *E*. *coli pho*A gene, which codes for the *E*. *coli* alkaline phosphatase, were ligated into transposon Tn*4001* in pISM2062.2, generating the pISM2062.2*ltufacypho*A plasmid (pTAP) [14].

#### Plasmid *ltuf*GTG*acypho*A (pTGP)

The ATG translational start codon of the reporter construct in the pTAP plasmid was mutated to GTG by overlap extension PCR. The *ltuf* promoter and the translational start codon were amplified as a 357 bp product by PCR using the IRF (5′ GGCCGgGATCAAGTCCGTATTATTGTGTAAAAGTgCtaGc 3′) and GTGR (5′ CTTTAAAATGTTTTTTCTCTTCAcTTTTTTAAATATTTCTCC 3′) oligonucleotide primers. Mutation of the first nucleotide of the start codon was achieved by incorporating this change in the GTGR primer.

The translational start codon, the *vlh*A1.1 signal sequence and the *pho*A coding sequence were amplified as a product of 1,479 bp from the pTAP plasmid using the oligonucleotide primers GTGF (5**′** GGAGAAATATTTAAAAAAgTGAAGAGAAAAAACATTTTAAAG 3**′**) and PBgR (5**′** CCGaGATctaAAAGGACTGttaTATGGCCTTTTTATTTTATTTCAGCCCCAGA 3**′**). The PCR products were purified after electrophoresis in a 1% agarose gel using the Qiaex gel extraction kit (Qiagen) and joined by overlap extension PCR using the primers IRF and PBgR, resulting in the 1,792 bp *ltuf*GTG*acypho*A product.

The resultant PCR product was gel purified and ligated into pGEM-T (Promega) following the manufacturer’s instructions. An *E*. *coli* transformant containing a plasmid of the expected size was selected and the insert DNA sequence confirmed using BigDye terminator v3.1 cycle sequencing (Perkin Elmer Applied Biosystems) and the M13 universal primers. The 1,620 bp DNA insert was released from pGEM-T by digestion with the restriction endonucleases *Nhe*I and *Sph*I, purified using the Qiaex gel extraction kit and ligated into similarly digested pTAP, resulting in pISM2062.2*ltuf*GTG*acypho*A (pTGP).

The plasmid pTGP was introduced into *E*. *coli* DH5α by electroporation using a Gene Pulser (Bio-Rad) with settings of 2.5 kV and 25 μF. Transformants were selected for ampicillin resistance and the clones were screened for the presence of the gentamicin resistance gene in the transposon by PCR using the oligonucleotide primers GmF and GmR, which yielded a 223 bp product [14]. Selected clones were cultured in larger volumes and plasmid DNA was extracted using a Midi prep kit (Qiagen) according to the manufacturer’s instructions. The DNA sequence of the pTGP plasmid was confirmed using BigDye terminator v3.1 cycle sequencing (Perkin Elmer Applied Biosystems) and the plasmid was then used to transform *M*. *gallisepticum* cells by electroporation and the colonies obtained picked and grown in mycoplasma broth containing gentamicin, as described previously [14]. The presence of the gentamicin resistance gene was confirmed by PCR using the oligonucleotide primers GmF and GmR.

### PCR

The thermal cycling conditions for amplification of DNA sequences by PCR and quantitative RT-PCR were as described previously [14].

### Insertion points of transposon constructs

To determine the insertion site of the Tn*4001* transposon, genomic DNA sequencing was carried out using the ABI Prism BigDye Terminator v3.1 sequencing system (Perkin Elmer Applied Biosystems) and the UBR oligonucleotide primer (5**′** GCAGTAATATCGCCCTGAGC 3**′**) [14].

### Detection of PhoA

Immunoblotting, localisation of PhoA (Triton X-114 fractionation, membrane fractionation and trypsin proteolysis) and alkaline phosphatase assays were performed as described previously [14].

## Results

### Analysis of translational start codons

The data on mycoplasma genomes were downloaded from the NCBI repository of completely sequenced bacterial genomes at ftp://ftp.ncbi.nih.gov/genomes/Bacteria/. The directory on the NCBI site was used to collate the information on start codons. GeneMark 2.5m was used to import the the sequence, G+C content and length of the sequence. The CDS data were obtained from the NC.rpt file. The start codon annotations from Prodigal 2.50 (Prokaryotic Dynamic programming Gene-finding Algorithm) [15] were downloaded and the count of the different start codons compiled. Prodigal detects only the 3 major start codons, AUG, GUG and UUG, with the non-standard codons AUU, AUA and CUG not considered. The tabulated frequencies of use of the different translational initiation codons in mycoplasmas are shown in Table 1. Based on these bioinformatic data, in mycoplasmas ATG was used in 87% of CDSs, GTG in 8% and TTG in 5%. In different *M*. *gallisepticum* strains, 85% of CDSs used ATG, 10% used GTG and 5% used TTG. The G+C content of the mycoplasma genomes varied from 24% to 40%. There was a modest correlation between G+C content and use of an ATG start codon (R2 = 0.4).

**Table 1:** Translational start codon usage in genomes of *Mollicutes*.

Species	Proportion (%) of CDSs predicted to use start codon:			% G+C	No. CDSs	Accession No.
	ATG	GTG	TTG			
*Acholeplasma laidlawii* PG-8A	92.58	2.88	4.54	31.08	1380	NC_010163.1
*Mycoplasma agalactiae* PG2	93.49	2.05	4.46	29.62	742	NC_013948.1
*Mycoplasma arthritidis* 158L3-1	94.32	4.10	1.58	30.71	631	NC_011025.1
*Mycoplasma bovis* Hubei-1	95.14	2.24	2.62	29.29	801	NC_015725.1
*Mycoplasma bovis* PG45	95.45	1.67	2.87	29.31	765	NC_014760.1
*Mycoplasma capricolum* subsp. *capricolum* ATCC 27343	96.19	2.74	1.07	23.77	812	NC_007633.1
*Mycoplasma conjunctivae* HRC/581	93.46	2.67	3.86	28.49	692	NC_012806.1
*Mycoplasma crocodyli* MP145	97.23	0.79	1.98	26.95	689	NC_014014.1
*Mycoplasma fermentans* JER	94.15	2.39	3.46	26.95	797	NC_014552.1
*Mycoplasma fermentans* M64	93.32	2.53	4.15	26.86	1050	NC_014921.1
*Mycoplasma gallisepticum* F	89.40	7.79	2.81	31.4	756	NC_017503.1
*Mycoplasma gallisepticum* R(high)	83.27	11.15	5.58	31.47	766	NC_017502.1
*Mycoplasma gallisepticum* R(low)	83.62	10.67	5.71	31.47	763	NC_004829.2
*Mycoplasma genitalium* G37	86.88	9.51	3.61	31.69	475	NC_000908.2
*Mycoplasma haemocanis* Illinois	80.19	13.38	6.44	35.33	1156	NC_016638.1
*Mycoplasma haemofelis* Ohio2	82.78	11.15	6.07	38.81	1527	NC_017520.1
*Mycoplasma haemofelis* Langford 1	83.35	11.22	5.42	38.85	1545	NC_014970.1
*Mycoplasma hominis* ATCC 23114	95.71	1.25	3.04	27.12	523	NC_013511.1
*Mycoplasma hyopneumoniae* 168	92.78	4.11	3.12	28.46	693	NC_017509.1
*Mycoplasma hyopneumoniae* 232	92.79	3.68	3.53	28.56	691	NC_006360.1
*Mycoplasma hyopneumoniae* 7448	93.50	3.61	2.89	28.49	657	NC_007332.1
*Mycoplasma hyopneumoniae* J	91.93	4.61	3.46	28.52	657	NC_007295.1
*Mycoplasma hyorhinis* GDL-1	89.51	2.92	7.57	25.91	647	NC_016829.1
*Mycoplasma hyorhinis* HUB-1	89.33	3.43	7.25	25.88	658	NC_014448.1
*Mycoplasma hyorhinis* MCLD	89.67	3.84	6.49	25.88	778	NC_017519.1
*Mycoplasma leachii* PG50	94.13	2.37	3.50	23.75	882	NC_014751.1
*Mycoplasma mobile* 163K	94.55	2.58	2.88	24.95	633	NC_006908.1
*Mycoplasma mycoides* subsp. *capri* LC 95010	96.73	2.64	0.63	23.82	922	NC_015431.1
*Mycoplasma mycoides* subsp. *mycoides* SC PG1	83.90	10.62	5.48	23.97	1017	NC_005364.2
*Mycoplasma penetrans* HF-2	95.86	2.79	1.35	25.72	1037	NC_004432.1
*Mycoplasma pneumoniae* 309	81.82	12.65	5.53	39.98	707	NC_016807.1
*Mycoplasma pneumoniae* FH	81.25	12.11	6.64	40.00	629	NC_017504.1
*Mycoplasma pneumoniae* M129	81.41	12.30	6.28	40.01	689	NC_000912.1
*Mycoplasma pulmonis* UAB CTIP	92.45	4.77	2.78	26.64	782	NC_002771.1
*Mycoplasma putrefaciens* KS1	92.86	5.00	2.14	26.94	650	NC_015946.1
*Mycoplasma suis* KI3806	65.00	10.00	25.00	31.08	794	NC_015153.1
*Mycoplasma suis* Illinois	67.17	10.06	22.77	31.08	845	NC_015155.1
*Mycoplasma synoviae* 53	90.14	4.08	5.77	31.08	659	NC_007294.1
*Ureaplasma parvum* serovar 3 ATCC 27815	92.27	3.45	4.28	31.08	609	NC_010503.1
*Ureaplasma parvum* serovar 3 ATCC 700970	91.92	3.39	4.68	31.08	614	NC_002162.1
*Ureaplasma urealyticum* serovar 10 ATCC 33699	91.82	3.48	4.70	31.08	646	NC_011374.1

The CDSs and the %G+C data were obtained from the NCBI site for bacterial genomes (ftp://ftp.ncbi.nih.gov/genomes/Bacteria/) and the start codon data compiled using Prodigal 2.50.

### Analysis of the codons surrounding the start codon

Analysis of the codons surrounding the start codon in 125 species of bacteria have deetcted a preference for lysine, serine or threonine as the amino acid after the start codon (2^nd^ amino acid). In firmicutes, lysine is the predominant second amino acid [16], and this was the 2^nd^ amino acid in the fusion protein encoded by both pTAP and pTGP. The presence of A/T rich codons around the start codon may reduce formation of secondary structures and favour higher rates of translational initiation [17]. Isoleucine is overrepresented in the fourth to eighth positions in 91% of the prokaryotic genomes that have been analysed, and this was also the case in the fusion protein studied here.

### Insertion points of transposon constructs

To confirm that the Tn*4001* transposon integrated at random in the genome the site of integration of the transposons from the pTGP plasmid in the *M*. *gallisepticum* genome was determined for three of the transformants (TGP2, TGP3, TGP5) by genomic DNA sequencing [14]. The annotated genome sequence of *M*. *gallisepticum* strain Rlow (NC_004829.2) was used as the reference genome to determine the location of the transposon. The transposon integrated into the *M*. *gallisepticum* genome at random, within lipoprotein (TGP2::MGA_0981), putative bacteriocin/lantibiotic ABC exporter (TGP5::MGA_0022) and hypothetical (TGP3::MGA_0471) genes. The proportion of the coding sequence of these genes upstream of the insertion point of the transposon varied from 40% to 86%.

### Levels of transcription of *pho*A in transformants

Three pTAP transformants (pTAP3, 4, 9) and three pTGP transformants (pTGP2, 3, 5) were selected and mRNA extracted to compare levels of transcription of the *pho*A gene by quantitative RT-PCR. The *pho*A mRNA abundance relative to GAPDH mRNA in each transformant was determined in triplicate, and the mean levels of transcription of *pho*A in the pTAP and pTGP transformants were compared. The levels of *pho*A mRNA (mean ± SEM) were determined in pTGP2 (4.8 ± 0.8), pTGP3 (6.4 ± 1.1), pTGP5 (3.5 ± 0.5), pTAP3 (12.5 ± 1.5), pTAP4 (10.9 ± 1.4) and pTAP9 (13.4 ± 1.5). Thus the relative abundances of *pho*A mRNA transcripts were similar in the clones transformed with the same construct and the mean level of *pho*A transcription in pTAP transformants (12.1 ± 0.7) was significantly higher than in pTGP transformants (4.9 ± 0.8) (P = 0.003, Student’s t-test).

### Alkaline phosphatase activity of pTGP-transformed *M*. *gallisepticum*

The level of alkaline phosphatase activity was determined in five randomly selected pTAP and pTGP transformants in triplicate as described previously [14]. The mean level (± SEM) of alkaline phosphatase activity for pTAP transformants was 190 ± 8 U/mg total cell protein, which was significantly higher than in pTGP transformants (119 ± 11 U/mg total cell protein) (P = 0.03, Student’s t-test).

### Efficiency of translational initiation codon

The relative efficiency of translation was determined by dividing alkaline phosphate activity by the relative *pho*A mRNA concentrations. The relative levels of translation were determined in the transformants pTGP2, pTGP3, pTGP5, pTAP3, pTAP4 and pTAP9 as shown in Table 2. The relative efficiency of PhoA translation in pTGP transformants (26.6 ± 1.4) was significantly higher than in pTAP transformants (16.7 ± 2.6) (P = 0.045, Student’s t-test).

**Table 2:** Efficiency of transcription and translation of *pho*A constructs with a GTG (TGP) or ATG (TAP) translational start codon in *M*. *gallisepticum* transformants

Transformant	Alkaline phosphatase (mean units/mg ± SEM)	mRNA (mean ± SEM)	Relative efficiency of translation (mean ± SEM)
TGP2	118.7 ± 11.3	4.8 ± 0.8	27.5 ± 8.5
TGP3	142.4 ± 18.2	6.4 ± 1.1	23.8 ± 4.8
TGP5	94.5 ± 3.9	3.5 ± 0.5	28.4 ± 5.7
TAP3	259.4 ± 5.9	12.5 ± 1.5	21.4 ± 2.9
TAP4	172.2 ± 7.8	10.9 ± 1.4	16.3 ± 1.9
TAP9	160.5 ± 1.2	13.4 ± 1.5	12.3 ± 1.4

### Localisation of PhoA fusion protein

**Fig 1:**
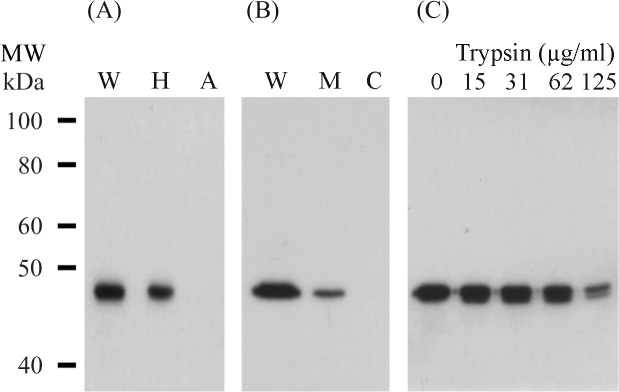
Localisation of PhoA proteins in M. gallisepticum cells. (A) Proteins of the pTGP3 transformant were separated into hydrophobic and aqueous fractions by Triton X-114 partitioning. Equivalent amounts of the fractions were separated by SDS-PAGE in 10% polyacrylamide gels, Western transferred and probed with a monoclonal antibody against alkaline phosphatase. Lanes: W, whole cells; H, hydrophobic fraction; A, aqueous fraction. The molecular weight (MW) markers were the biotinylated protein ladder from Cell Signaling Technology. (B) The membrane and cytosolic fractions of transformant pTGP3 were separated by SDS-PAGE in a 10% polyacrylamide gel and Western transferred. Immunostaining with a monoclonal antibody to alkaline phosphatase demonstrated the presence of PhoA in the whole cells (lane W) and the membrane fraction (lane M), but not in the cytosolic fraction (lane C). (C) Whole cells of the pTGP3 transformant were treated with increasing concentrations of trypsin. The cells in lane 0 were treated with TS buffer only. Proteins were separated by SDS-PAGE in 10% polyacrylamide gels and immunostained with a monoclonal antibody against alkaline phosphatase.

Whole cell proteins of the pTGP3 transformant were subjected to Triton X-114 fractionation and the protein fractions were separated by SDS-PAGE, transferred to polyvinylidene fluoride (PVDF) membranes and immunostained using a monoclonal antibody against alkaline phosphatase [14]. A band of 47 kDa, corresponding to the predicted molecular weight of expressed PhoA fusion protein, was detected in pTGP3-transformed *M*. *gallisepticum* whole cell proteins (Fig 1A, lane W) and in the hydrophobic fraction (Fig 1A, lane H), but not in the aqueous fraction (Fig 1A, lane A).

The pTGP3 transformant was separated into membrane and cytosolic fractions by differential ultracentrifugation [14]. The proteins were separated by SDS-PAGE, transferred to PVDF membranes and immunostained. The PhoA fusion protein was detected in the membrane fraction (Fig 1A, lane M), and also in whole cells (Fig 1A, lane W), but not in the cytosolic fraction (Fig 1A, lane C).

The surface exposure of PhoA in the pTGP3 transformant was examined by trypsin proteolysis [14]. Immunostaining of trypsin-treated cells with a monoclonal antibody against alkaline phosphatase demonstrated a gradual loss of reactivity with increasing concentrations of trypsin (Fig 1C), indicating surface exposure of the PhoA fusion protein.

## Discussion

There was a 2.5 fold decrease in transcription of the *ltufacypho*A gene when the GTG start codon was used. As the nucleotides in the untranslated leader region (UTR) were not mutated, a possible factor influencing transcription could be the mRNA secondary structure. The minimal free energy for optimal secondary structure was higher with the ATG codon (-2.5 kCal mol^-1^), which could have reduced the stability of the mRNA secondary structure and increased transcriptional efficiency. A significantly higher free energy for genes with an ATG initiation codon has been reported in *B*. *subtilis* and it is possible that start codon preference is partly influenced by mRNA structure [11]. In *B*. *subtilis*, different start codons affect the stability of the Δ*ermC* mRNA, with mutation of the start codon from ATG to GTG resulting in a significant decrease in the mRNA half-life, from 8.2 min to 6.5 min, which has been putatively attributed to an effect of ribosomal binding and ternary complex formation on stability [18]. In *E*. *coli* mRNA levels are highly correlated with folding energy near the 5’ end of the transcript. The secondary structure can obstruct the binding of the ribosomal subunit and thus translational initiation. When there is reduced ribosomal binding, mRNA is also exposed to nuclease digestion [19]. Mazin *et al* have recently described an experimental genome-wide study of regulation of transcription in *M*. *gallisepticum* strain S6, and detected transcriptional changes under different stress conditions. The A/T content, the first nucleotide of the transcript and the spacer distance appeared to correlate with variations in transcription [20].

It has been suggested that when GTG replaces the ATG codon there is less efficient pairing with the fMet-tRNA, which can reduce the rate of translational initiation [21]. A 3 to 8 fold reduction in protein expression is seen with a GTG start codon in *B*. *subtilis* [12, 22] and *E*. *coli* [10, 12, 23, 24]. While alteration of the start codon of the *pho*A gene in the pTAP plasmid from ATG to GTG resulted in reduced levels of expression of alkaline phosphatase in *M*. *gallisepticum*, the reduction in expression was considerably less than the reduction in transcription of the gene.

The relative efficiency of translation was determined for the ATG and GTG initiation codons. The GTG codon appeared to result in a significantly higher relative efficiency of translation (26.6 ± 1.4) than the ATG codon (16.7 ± 2.6), suggesting that it was a more efficient translational initiation codon. While a relatively small number of transformants generated with each construct were evaluated, the within-transformant variation was similar to the between-transformant variation for the three pTGP and three pTAP transformants evaluated. Furthermore, there was a significant difference in levels of transcription, expression and translational efficiency between the transformants with the different start codons in the construct. Therefore the small number of transformants was sufficient for effective analysis.

The *phoA* reporter gene used in this study was fused to the lipoprotein signal sequence of a *vlhA* gene, that we have previously shown results in translocation of the protein through the cytoplasmic membrane, acylation of the protein and exposure on the surface of the mycoplasma cell. In order to confirm that there was not a differential effect of the two alternative start codons on the processing of the lipoprotein reporter, we examined the localization of the expression products of the two constructs. Partitioning of cellular proteins into the hydrophobic and hydrophilic fractions with Triton X-114 demonstrated that with both constructs the PhoA fusion protein was within the hydrophobic fraction, and cell surface proteolysis showed that it was also sensitive to trypsin. This confirmed that the PhoA fusion protein was exported to the membrane and surface exposed, suggesting that lipoprotein processing and export was similar with both ATG [14] and GTG start codons.

The native *tuf* gene has an ATG start codon, while the native *vlh*A 1.1 gene has a GTG start codon. It is not clear if the sequence of the promoter region may have played a role in the higher levels of transcription seen with the ATG codon in the pTAP transformants. It is possible that the reduced translational efficiency of the gene containing the GTG codon may have directly influenced the efficiency of transcription of the gene and it is also possible that there is not a linear correlation between transcript abundance and translation.

The mean usage of the three major initiation codons across 620 bacterial chromosomes is 80.1% for ATG, 11.6% for GTG and 7.8% for TTG, with low G+C genomes showing a greater bias towards use of the ATG codon [9]. Mycoplasmas deviate from the universal genetic code in using TGA as a codon for tryptophan [25, 26], which is presumed to be a result of directional mutational pressure during evolution towards an A+T rich genome. The predominant translational initiation codon based on *in silico* prediction of coding sequences in *Mycoplasma* spp. is ATG (Table 1), as might be expected in organisms with a low genomic G+C content. However, the order of preference of ATG > GTG > TTG is more similar to the pattern seen in *E*. *coli* than to that seen in *B*. *subtilis* [11], and does not appear to concord with the strong bias in mycoplasmas towards codons with a low G+C content. These results are primarily based on genome annotation and could change with experimental analysis. In *M*. *pneumoniae* using proteomic approaches two novel genes were identified and one translational start codon was corrected [27], while in *Mycoplasma mobile* both genomic and proteogenomic mapping by mass spectrometry were combined to guide annotation of the genome [28]. In future such combinations of proteogenomic approaches may lead to correction of genome annotation errors, and identification and validation of additional or alternative translational start codons in the genome [29].

The transcriptional and translational regulation mechanisms and signals in mycoplasmas are considered similar, but not identical, to those seen in other bacteria [30]. In *M*. *pneumoniae* there is only a modest correlation between mRNA and protein abundance, and it has been suggested that translational regulation may be more important in mycoplasmas than regulation of transcription [31]. While the much smaller difference in the effect of a differing start codon on the efficiency of expression in *M*. *gallisepticum* compared to that seen in other bacteria can explain why there is not a strong selection against use of a GTG start codon, it is not clear why there is such a strong preference for its use in a specific gene family. Further studies on regulation of transcription and translation in mycoplasmas will be necessary to establish whether the GTG start codon plays a role in translational regulation of protein expression that was not elucidated in the studies we have described here.

In conclusion the ATG start codon is the preferred start codon for translation in most mycoplasma species. In this study, using the pTAP construct, GTG was shown to be a more efficient translational initiation codon in *M*. *gallisepticum*, although less efficient transcription in constructs using this start codon resulted in lower overall levels of expression. This finding contrasts with observations of reduced protein expression when ATG start codons were replaced with GTG in *E*. *coli* and *B*. *subtilis*, suggesting there may be significant differences in the mechanisms involved in translational initiation and regulation in mycoplasmas.
